# Benefits from Shortening Viral Shedding by Traditional Chinese Medicine Treatment for Moderate COVID-19: An Observational Study

**DOI:** 10.1155/2022/7179050

**Published:** 2022-02-01

**Authors:** Qiliang Zhao, Lei Zhang, Shuo Zhang, Xiaoxue Gao, Yuye Li, Minghu Chen, Xiumei Gao, Min Liu

**Affiliations:** ^1^First Teaching Hospital of Tianjin University of Traditional Chinese Medicine, Tianjin, China; ^2^National Clinical Research Center for Chinese Medicine Acupuncture and Moxibustion, Tianjin, China; ^3^Tianjin Hospital, Tianjin, China; ^4^Second Affiliated Hospital of Tianjin University of Traditional Chinese Medicine, Tianjin, China; ^5^Tianjin University of Traditional Chinese Medicine, Tianjin, China; ^6^Binhai New Area Hospital of Traditional Chinese Medicine, Fourth Teaching Hospital of Tianjin University of Traditional Chinese Medicine, Tianjin, China

## Abstract

Traditional Chinese medicine (TCM) treatment for the coronavirus disease 2019 (COVID-19) can improve clinical symptoms, but it is not clear whether it can shorten viral shedding. This is an observational study including 97 patients with COVID-19 who were consecutively admitted to the Jiangxia Fangcang hospital in Wuhan (Hubei, China) from January 15, 2020, to March 10, 2020. All patients were treated with TCM, and we assessed the patients daily and collected clinical information via a diary card. The primary endpoint was the time to achieve a negative result for severe acute respiratory syndrome-coronavirus 2 (SARS-CoV-2) RT-PCR. The final analysis included 92 patients. The median time to negative oropharyngeal swab for all the participants was 22 days (IQR 15–30). The participants were divided into three groups according to time from symptom onset to start of TCM treatment: within 7 days group (early treatment group), 8–14 days group (middle treatment group), and over 14 days group (late treatment group). The median time to negative oropharyngeal swab for the early treatment group was 14 days (IQR 12–17) and for the middle and late treatment groups was statistically shorter than 20 days (IQR 18–22) and 30 days (IQR 25–34), respectively. In univariate Cox proportional hazards regression analysis, the incidence of negative oropharyngeal swab for the early and middle treatment groups was 7.674 times and 3.609 times statistically higher than the late treatment group, respectively; whereas in multivariate Cox proportional hazards regression analysis, the incidence for the early and middle treatment groups was 18.093 times and 5.804 times statistically higher than the late treatment group, respectively. In patients with moderate COVID-19, those who had no cough, no dyspnea, and those who received TCM treatment earlier could achieve nucleic acid negative sooner by shortening viral shedding.

## 1. Introduction

The COVID-19 pandemic caused by the SARS-CoV-2 has affected more than 252 million patients, with more than 5.09 million deaths in 228 countries and regions [1]. The pandemic is still spreading and remains a threat to human health.

Although many clinical trials of drugs have yielded preliminary results, the recognized effective treatment for COVID-19 remains uncertain. In clinical trials of antiviral drugs, remdesivir was used in the treatment of severe COVID-19 cases, but the findings are inconclusive. A study showed that 68% of patients reached clinical improvement in terms of oxygen-support status, and the most common adverse events were diarrhea, rash, renal impairment, hypotension, and increased hepatic enzymes [2]. However, a clinical trial in China showed that remdesivir was not associated with statistically significant clinical benefits [3]. On May 1, 2020, the FDA provided an Emergency Use Authorization (EUA) for remdesivir, and it is the only medication that has been approved for COVID-19 infection by the U.S. Food and Drug Administration so far. Lopinavir/ritonavir has been reported to lower the viral load, mortality rates, and adverse respiratory distress syndrome (ARDS) in the treatment of severe COVID-19 cases, but it lacks statistical support [4, 5]. Another controlled clinical study of favipiravir (FPV) versus lopinavir (LPV)/ritonavir (RTV) for the treatment of COVID-19 showed that the virus clearance time of FPV group was shorter than that of LPV/RTV group (4d versus 11d, *P* < 0.001) [6]. The potential effects of antiviral drugs still require further large sample studies.

Treatment of COVID-19 using TCM can result in marked improvement in symptoms and shortened disease course [7–10]. Based on the TCM treatment experience in SARS, application of TCM in the treatment of COVID-19 is largely recommended. The TCM scheme was included in the guideline on diagnosis and treatment of COVID-19 in China [11]. Currently, a total of 60,107 confirmed cases have been treated with TCM. Treatment with TCM can shorten the clinical symptoms disappearance time (2 days), recovery time of body temperature (1.7 days), and the average length of hospital stay (2.2 days). It can also improve the rate of computed tomography (CT) image improvement (22%) and clinical cure rate (33%), reduce the rate of common to severe cases (27.4%), and increase the lymphocyte count (70%). In addition, the effective cure rate of Qingfei Paidu decoction against COVID-19 is over 90% [12]. Clinical practice has proven that early adopt TCM scheme to treat COVID-19 can improve cure rate, shorten disease course, delay disease progression, and reduce mortality rate [13].

Until now, it is not clear whether TCM treatment influences shortening of the viral shedding. Thus, the objective of this study is to evaluate the effects of TCM on shortening viral shedding.

## 2. Materials and Methods

### 2.1. Study Design

An observational study including consecutive COVID-19 patients was performed in the Jiangxia Fangcang hospital of Wuhan (Hubei, China) from January 15, 2020, to March 10, 2020.

### 2.2. Patients

Eligible patients were men with COVID-19 who were aged at least 16 years and were RT-PCR positive for SARS-CoV-2 and had pneumonia confirmed by chest imaging. Patients were diagnosed as moderate COVID-19 according to the COVID-19 treatment guidance (Seventh Edition of China). Patients only received TCM treatment for COVID-19, and the treatment of the coexisting diseases is uninterrupted. Exclusion criteria included age >85 years and severe and critically ill patients, including but not limited to the following: after cardiopulmonary resuscitation; patients with other organ failure or conditions requiring intensive care unit (ICU) monitoring and treatment, such as severe liver disease, severe renal dysfunction, and septic shock; or respiratory failure that needs mechanical ventilation.

### 2.3. Procedures

After enrollment, all participants received Chinese herbal decoction (named Anti-SARS-CoV-2 No.2) with no antiviral drugs throughout the hospitalization. The TCM prescription contains 11 herbs: gualoupi (Pericarpium Trichosanthis), 15 g; banxia (Rhizoma Pinelliae), 10 g; huangqin (Radix Scutellariae), 6 g; zhibaibu (honey-fried Herbs Radix Stemona), 10 g; qianhu (Radix Peucedani), 10 g; baiqian (Rhizoma Cynanchum Stauntonii), 10 g; ziwan (Radix Asteris), 10 g; chenpi (Pericarpium Citri Reticulatae), 10 g; gancao (Radix Glycyrrhizae), 10 g; jiegeng (Radix Platycodonis), 10 g; and jingjie (Herba Schizonepetae), 10 g.  Table 1 lists the names of these herbs in Chinese, Latin, and English. The treatment of the coexisting diseases—cardiovascular, cerebrovascular, and metabolic diseases—is uninterrupted, such as the use of antihypertensive drugs and antidiabetic drugs.

**Figure 1:**
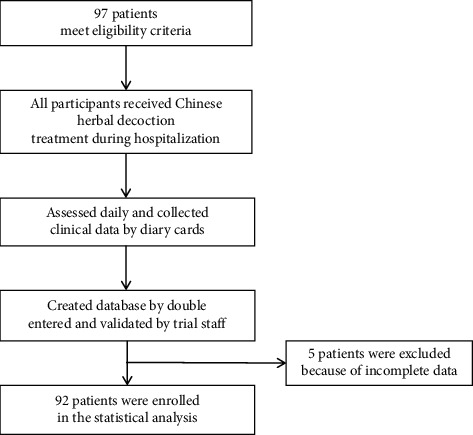
Trial profile.

Patients were assessed once daily by trained doctors using diary cards. Clinical data were recorded on paper case record forms and then double entered to an electronic database by EXCEL software and validated by trial staff. Oropharyngeal swab specimens were obtained 48 hours after remission of clinical symptoms, and patients with two negative oropharyngeal swab RT-PCR within 24 hours could be discharged. The trial profile is shown in Figure 1.

### 2.4. Outcomes

The primary clinical endpoint was time from start of TCM treatment to a negative RT-PCR result for SARS-CoV-2 in an oropharyngeal swab sample. The negative oropharyngeal swab RT-PCR was defined as testing negative for two consecutive tests.

### 2.5. Statistical Analysis

The time from start of TCM treatment to a negative RT-PCR was portrayed using the Kaplan–Meier plot and compared with a log-rank test. The hazard ratio (HR) and 95% confidence interval (CI) of candidate predictors for the negative viral load was calculated by the Cox proportional hazards model. Subgroup analysis was performed according to the characteristics of the patients. SPSS software 21.0 and SAS software 9.4 were used for statistical analyses. A *P* value of less than 0.05 was considered statistically significant.

## 3. Results

From January 15, 2020, to March 10, 2020, 97 patients from the Jiangxia Fangcang hospital in the Hubei province were enrolled; five of these patients were excluded because of incomplete data. A total of 92 patients were included in the statistical analysis. Of these patients, all were male (100%) because the hospital only accepted male patients. The age range was 17 to 68 years, while the majority (51%) were aged between 40 and 60 years. The most common comorbidity was cardiovascular and cerebrovascular diseases (20.7%), including hypertension in 18 patients and coronary heart disease in two patients. This is followed by metabolic diseases (6.5%), including diabetes in four patients, hyperuricemia in two patients, and chronic hepatitis B in three patients (3.3%). There were 30 patients in the early treatment group (32.6%), 21 patients in the middle treatment group (32.6%), and 41 patients in the late treatment group (44.6%). The number of patients with fever as the major complaint at enrollment was 47 (51.1%), 42 presented with cough (45.7%), dyspnea in 12 (13.0%), respiratory symptoms in 47 (51.1%), fever with respiratory symptoms in 17 (18.5%), and fatigue in 7 (7.6%). Sixty-three (68.5%) patients presented with multilobar and 29 (31.5%) patients presented with single lobar distribution on chest CT imaging. Eighty-five (92.4%) patients presented with ground-glass opacity (GGO) and seven (7.6%) presented with consolidation on chest CT imaging. Ten (10.9%) patients presented with diffuse and 82 (89.1%) patients presented with patchy distribution on chest CT imaging. There were four (4.3%) patients who presented with decreased white blood cell count, and 88 (95.7%) patients presented with normal white blood cell count. Seven (7.6%) patients presented with decreased lymphocyte count, and 85 (92.4%) patients presented with normal lymphocyte count. Lastly, 11 (12.0%) patients presented with C-reactive protein elevation, and 81 (88.0%) patients presented with normal values.

**Figure 2:**
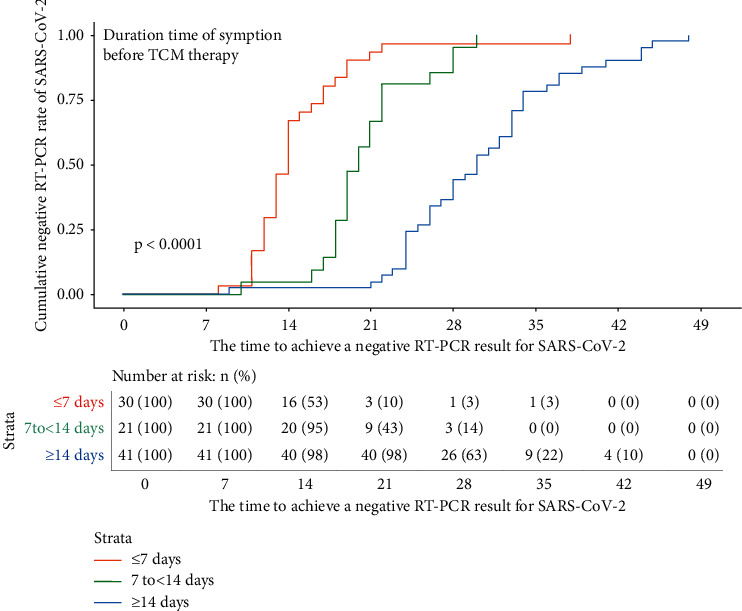
Kaplan–Meier survival curve of the influence of duration time of symptoms before TCM therapy on the negative oropharyngeal swab RT-PCR time of SARS-CoV-2.

**Figure 3:**
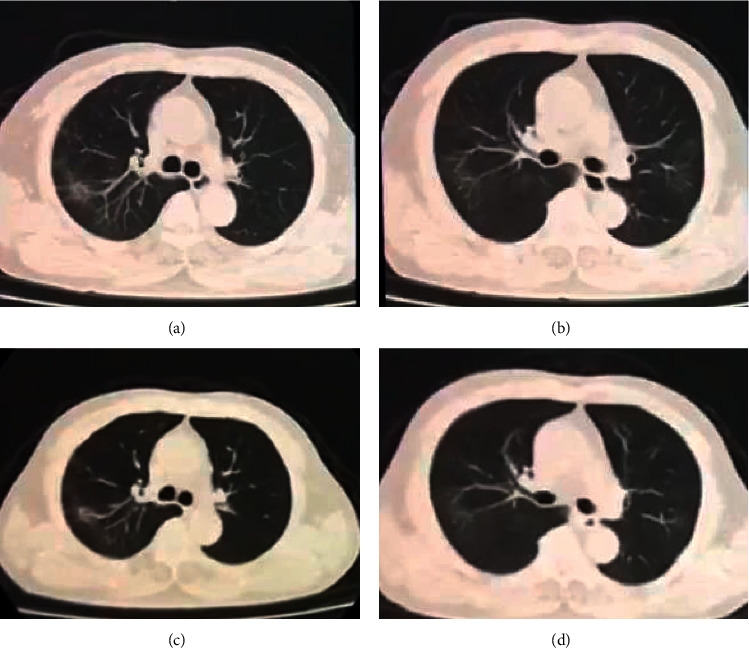
A 61-year-old male patient with cough and expectoration 6 days before TCM treatment, the time from start of TCM treatment to negative oropharyngeal swab is 13 days. (a, b) Chest CT presented with GGOs in the area of right upper lobe and left upper lobe before TCM treatment. (c, d) Follow-up chest CT scans taken 7 days after TCM treatment showing partial absorption of GGOs.

**Figure 4:**
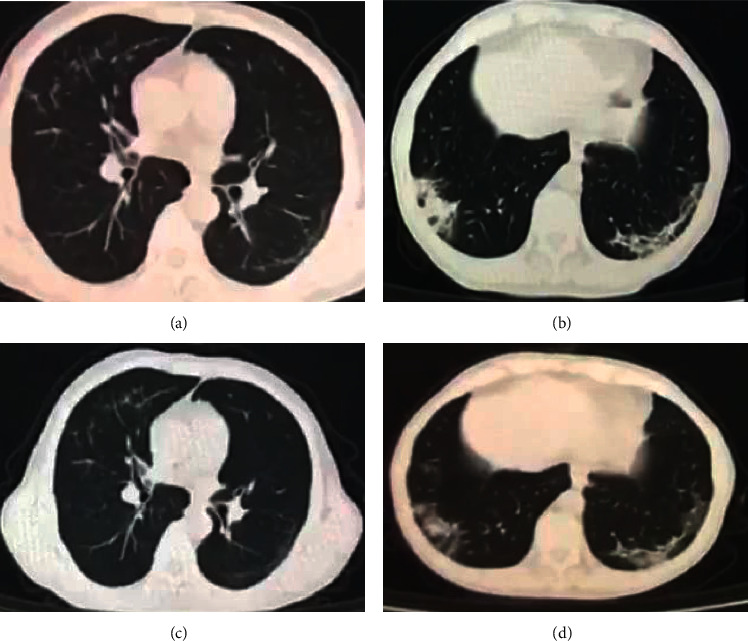
A 58-year-old male patient with fatigue and palpitation 12 days before TCM treatment, the time from start of TCM treatment to negative oropharyngeal swab is 28 days. (a, b) Chest CT presented with multipatchy, GGOs, and light consolidation in the peripheral area before TCM treatment. (c, d) Follow-up chest CT scans taken 7 days after TCM treatment showing partial absorption of all lesions.

**Figure 5:**
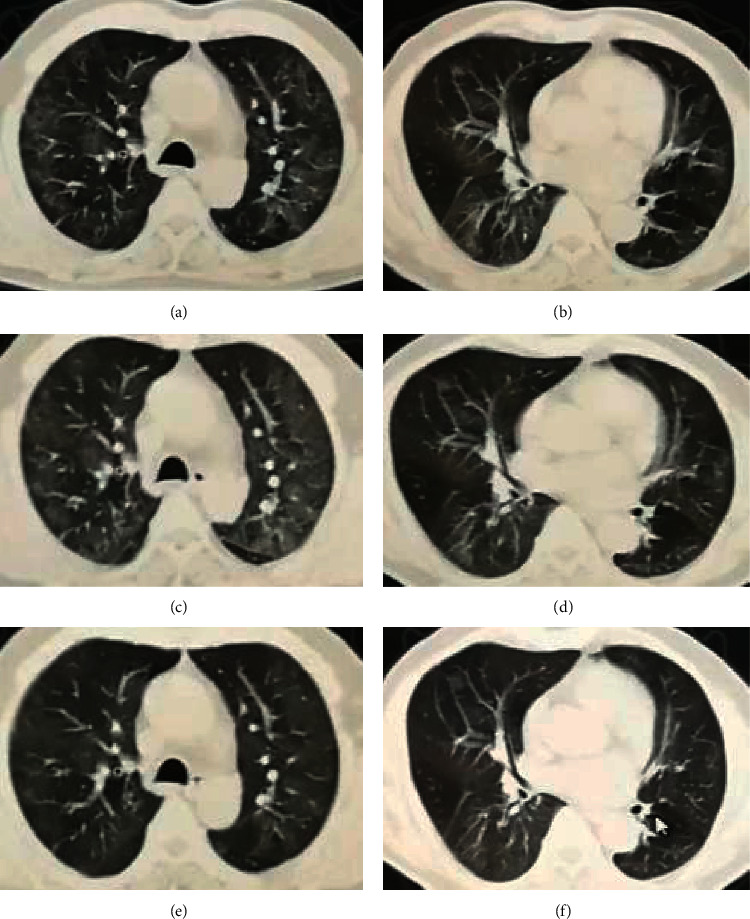
A 63-year-old male patient with fever and cough 20 days before TCM treatment, the time from start of TCM treatment to negative oropharyngeal swab is 32 days. (a, b) Chest CT presented with diffuse GGOs before TCM treatment. (c, d) Follow-up chest CT scans taken 7 days after TCM treatment showing the GGOs is more diffuse than before and the disease is still progressing. (e, f) Follow-up chest CT scans taken 14 days after TCM treatment showing partial absorption of GGOs.

For the primary endpoint of duration from start of TCM treatment to a negative viral RT-PCR result for SARS-CoV-2, the Kaplan–Meier survival analysis was used for subgroup analysis of different factors. The median duration from start of TCM treatment to a negative viral RT-PCR according to age was as follows: 11 days for age ≤20 years (IQR 10–26), 21 days for age 21–40 years (IQR 16–30), 22 days for age 41–60 years (IQR 17–28), and 26 days for age >60 years (IQR 13–32), but the difference was not statistically significant (*P*=0.831). When stratified by the coexisting cardiovascular and cerebrovascular diseases, metabolic disease, and chronic hepatitis B, the median duration from start of TCM treatment to a negative viral RT-PCR with and without the above coexisting conditions was 21 days vs 22 days (IQR 14–32 VS 16–28), 24 days vs 22 days (IQR 19–32 VS 14–30), and 33 days vs 22 days (IQR 28–34 VS 15–29), respectively, but the difference was not statistically significant (*P* = 0.951, 0.700, and 0.303). When stratified by duration from symptom onset to start of TCM treatment, the participants were divided into three groups: early, middle, and late treatment groups. The median time to negative oropharyngeal swab for the three groups was 14 days (IQR 12–17), 20 days (IQR 18–22), and 30 days (IQR 25–34), respectively. The Kaplan–Meier survival curve is shown in Figure 2. When stratified by chief complaint at enrollment, the median duration was 24 days for fever (IQR 19–30), 21 days for cough (IQR 15–33), 24 days for dyspnea (IQR 16–33), 22 days for respiratory symptoms (IQR 14–33), 24 days for fever with respiratory symptoms (IQR 19–33), and 26 days for fatigue (IQR 20–30), but the difference was not statistically significant. When stratified by chest CT imaging, the median duration with multilobar findings was 22 days (IQR 17–30), 21 days for single lobar findings (IQR 14–26), 22 days for GGO (IQR 14–30), 24 days for consolidations (IQR 21–28), 30 days for diffuse form (IQR 18–39)), and 21 days for patch form (IQR 14–28), but the difference was not statistically significant. The typical chest CT imaging features are shown in Figures 3–5. When stratified by blood cell count, the median duration was 13 days for decreased WBC, 22 days for normal WBC (IQR 16–30), 24 days for decreased lymphocyte (IQR 18–30), and 22 days for normal lymphocyte (IQR 15–30), but the difference was not statistically significant. When stratified by C-reactive protein, the median durations with elevated CRP was 19 days (IQR 13–28) and 22 days for normal CRP (IQR 16–30), but the difference was not statistically significant (Table 2).

In the univariate Cox proportional hazards regression analysis, when we consider age as a risk factor, the age more than 60 years was set as the reference. The incidences of negative viral RT-PCR in the population aged ≤20 years (HR 1.305, 95% CI, 0.48–3.57, *P*=0.604), aged 21–40 years (HR 1.316, 95% CI, 0.69–2.50, *P*=0.403), and aged 41–60 years (HR 1.235, 95% CI, 0.70–2.17, *P*=0.462) were all higher than reference, but the difference was not statistically significant. When we consider coexisting conditions as a risk factor, the incidence of negative viral RT-PCR without cardiovascular and cerebrovascular diseases (HR 0.985, 95% CI, 0.59–1.64, *P*=0.952) was lower than that with patients with coexisting conditions, while the incidence in patients without metabolic disease (HR 1.170, 95% CI, 0.51–2.68, *P*=0.712) was higher than patients without coexisting conditions; the difference was not statistically significant. When we consider duration from symptom onset to start of TCM treatment as a risk factor, the late treatment group was set as the reference. The incidence of negative oropharyngeal swab for the early treatment group was 7.674 times statistically higher than the reference (HR 7.674, 95% CI, 4.49–13.11, *P* < 0.001), and the incidence in the middle treatment group was 3.609 times statistically higher than the reference (HR 3.609, 95% CI, 2.02–6.45, *P* < 0.001). When we consider the chief complaint at enrollment as a risk factor, the incidences of negative oropharyngeal swab RT-PCR without fever (HR 1.202, 95% CI, 0.79–1.83, *P*=0.389), without cough (HR 1.423, 95% CI, 0.93–2.19, *P*=0.108), without dyspnea (HR 1.600, 95% CI, 0.86–2.97, *P*=0.137), without respiratory symptoms (HR 1.501, 95% CI, 0.97–2.33, *P*=0.071), and without fatigue (HR 1.618, 95% CI, 0.70–3.74, *P*=0.261) were all higher than the reference, but the differences were all not statistically significant. Considering the chest CT image as a risk factor, the multilobar distribution was set as the reference. The incidence of negative viral RT-PCR with single lobar (HR 1.240, 95% CI, 0.80–1.93, *P*=0.342) was higher than the reference. If GGO was set as the reference, the incidence of negative viral RT-PCR with consolidation (HR 1.011, 95% CI, 0.47–2.20, *P*=0.978) was higher than the reference. When diffuse form was set as reference, the incidence of negative viral RT-PCR with patch (HR 1.704, 95% CI, 0.83–3.32, *P*=0.117) was higher than the reference, but the differences were all not statistically significant. When we consider blood cell count as a risk factor, the decreased WBC was set as reference. The incidence of negative viral RT-PCR with normal WBC (HR 1.405, 95% CI, 0.51–1.41, *P*=0.510) was higher than the reference. When decreased lymphocyte was set as reference, the incidence of negative viral RT-PCR with normal lymphocyte (HR 1.092, 95% CI, 0.50–2.38, *P*=0.824) was higher than the reference, but the differences were not statistically significant. When we consider C-reactive protein as a risk factor, the elevated CRP was set as reference. The incidence of negative viral RT-PCR with normal CRP (HR 1.489, 95% CI, 0.79–2.83, *P*=0.223) was higher than the reference, but the difference was not statistically significant.

We selected factors that were significantly different in the univariate Cox proportional hazards regression analysis, and the factors that have clinical significance were put into multivariate Cox proportional hazards regression analysis. The results revealed that the early treatment group was 18.093 times statistically higher than the reference (HR 18.093, 95% CI, 8.48–38.59, *P* < 0.001) and the middle treatment group was 5.804 times statistically higher than the reference (HR 5.804, 95% CI, 3.00–11.23, *P* < 0.001). Patients without cough or dyspnea have statistically shorter time to a negative oropharyngeal swab and 4.101 times (HR4.101, 95% CI, 1.36–12.39, *P*=0.012) and 4.71 times (HR 4.71, 95% CI, 1.80–10.12, *P*=0.001) faster than with symptoms, respectively (Table 3).

## 4. Discussion

According to the results of this study, we hypothesized that treatment with TCM for patients with moderate COVID-19 might suppress the high initial viral load and reduce the time to a negative RT-PCR result for SARS-CoV-2 by shortening virus shedding. The negative RT-PCR result for SARS-CoV-2 by virus shedding may indicate disease recovery and noncontagious patients.

Clinical recovery and virus shedding are not always consistent. A study of oseltamivir used to treat influenza (within 5 days of symptom onset) showed that the PCR positive rate of influenza virus was 44% on the 7th day in the oseltamivir treatment group, but the median duration of major symptoms was only 3 days. The alleviation of clinical symptoms was earlier than the time to achieve a negative viral load [14]. Interferon beta-1b, lopinavir-ritonavir, and ribavirin were used in combination to treat COVID-19, and the result showed that the M-time to resolution of symptoms was 4 days, but the M-time of negative RT-PCR was 8 days. The alleviation of clinical symptoms was also earlier than the time to achieve a negative RT-PCR [15], which is consistent with our results.

The time to a negative RT-PCR result for SARS-CoV-2 by shortening the duration of viral shedding between mild to moderate and severe COVID-19 is not always consistent. The M-time to negative viral load was eight days in the triple combination treatment of patients with mild to moderate COVID-19 [15]. The accumulated rate of undetectable viral RNA was 50.4% on the 7th day in adults with severe COVID-19 treatment with remdesivir [3]. In our study, the median time to achieve a negative viral load was 22 days. This may be the result of the absence of antiviral drugs in our study. TCM has the advantage of improving clinical symptoms, but its relation to antiviral effect is not clear. TCM may have limited the antiviral effect, but the antiviral effect is not the only mechanism of TCM against SARS-CoV-2.

The duration of viral shedding was related to antiviral drugs on one hand and neutralizing antibodies (Nabs) on the other hand. Humoral response plays a key role in antiviral by regulating the production of antibodies. The plasma from convalescent patients of COVID-19 has been used to combat SARS-CoV-2. Polyclonal NAbs could be induced in some convalescent patients and will be effective in treating SARS-CoV-2 [16]. These NAbs can provide passive immune responses to viral infection, but the outcomes of passive plasma therapy are unpredictable due to the variability of sera in different patients [17]. The antibody-mediated humoral response and inflammatory response is the main mechanism of SARS-CoV-2 infections [17], but it is not clear whether this study adopted this mechanism. Thus, more studies are needed to confirm the mechanisms of TCM treatment for COVID-19.

Our study showed that the earlier the TCM treatment is begun, the sooner the negative viral load can be achieved. Early treatment with TCM is appropriate for the treatment of COVID-19 because TCM might suppress the high initial viral load and reduce the duration from high viral load to reach early relief of clinical symptoms. The viral load is usually associated with clinical symptoms, the higher the viral load, the more severe the clinical symptoms [18, 19]. This is the reason why patients without cough or dyspnea can significantly shorten the viral shedding.

Our study has several limitations. First, our study population is all male patients and the effect of gender on viral shedding is unclear. Second, severe cases were not included in our study, so the conclusion of this study cannot be extended to severe cases.

## 5. Conclusions

In patients with moderate COVID-19, those who had no cough, no dyspnea, and those who received TCM treatment earlier could achieve nucleic acid negative sooner by shortening viral shedding.

## Figures and Tables

**Table 1:** Chinese simplified script and traditional script and English translations of traditional Chinese medicines mentioned in this article.

English translation	Latin translation	Chinese simplified script	Traditional script
Gualoupi	Pericarpium Trichosanthis	瓜蒌皮	瓜蔞皮
Banxia	Rhizoma Pinelliae	半夏	半夏
Huangqin	Radix Scutellariae	黄芩	黃芩
Zhibaibu	Honey-fried Herbs Radix Stemona	炙百部	炙百部
Qianhu	Radix Peucedani	前胡	前胡
Baiqian	Rhizoma Cynanchum Stauntonii	白前	白前
Ziwan	Radix Asteris	紫菀	紫菀
Chenpi	Pericarpium Citri Reticulatae	陈皮	陳皮
Gancao	Radix Glycyrrhizae	甘草	甘草
Jiegeng	Radix Platycodonis	桔梗	桔梗
Jingjie	Herba Schizonepetae	荆芥	荊芥

**Table 2:** Patient characteristics and Kaplan–Meier estimate for difference in median time from start of TCM treatment to achieve a negative RT-PCR for SARS-CoV-2.

Characteristic		*n* (%) Total (*n* = 92)	Median days of negative RT-PCR *M* (IQR)		95% CI	*P* value
Age (median, yr)		49 (17–68)				
≤20		5 (5.4%)	11 (10–26)		8.85–13.15	0.831
21–40		23 (25.0%)	21 (16–30)		17.87–24.13	
41–60		47 (51.1%)	22 (17–28)		19.32–24.68	
>60		17 (18.5%)	26 (13–32)		17.05–34.95	
Sex		Male (100%)				
Coexisting conditions		34 (37.0%)				
Cardiovascular and cerebrovascular diseases		19 (20.7%)	No	22 (16–28)	19.77–24.23	0.951
			Yes	21 (14–32)	13.54–28.47	
Metabolic disease		6 (6.5%)	No	22 (14–30)	19.26–24.72	0.700
			Yes	24 (19–32)	13.20–34.80	
Chronic hepatitis B		3 (3.3%)	No	22 (15–29)	19.96–24.04	0.303
			Yes	33 (28–34)	25.00–41.00	
Duration time from symptom onset to start of TCM treatment, days						
*n* ≤ 7		30 (32.6%)	14 (12–17)		13.08–14.92	<0.001
7 < *n* ≤ 14		21 (22.8%)	20 (18–22)		18.52–21.48	
*n* > 14		41 (44.6%)	30 (25–34)		26.25–33.76	
Chief complaint at enrollment						
Fever		47 (51.1%)	24 (19–30)		21.13–26.87	0.369
Cough		42 (45.7%)	21 (15–33)		18.28–23.72	0.093
Dyspnea		12 (13.0%)	24 (16–33)		8.72–39.28	0.117
Respiratory symptoms		47 (51.1%)	22 (14–33)		18.66–25.34	0.059
Fever with respiratory symptoms		17 (18.5%)	24 (19–33)		18.96–29.04	0.265
Fatigue		7 (7.6%)	26 (20–30)		20.87–31.13	0.237
Chest CT						
Distribute	Multilobar	63 (68.5%)	22 (17–30)		18.54–25.46	0.321
	Single	29 (31.5%)	21 (14–26)		15.73–26.27	
Density	GGO	85 (92.4%)	22 (14–30)		18.40–25.60	0.977
	Consolidation	7 (7.6%)	24 (21–28)		21.66–26.34	
Form	Diffuse	10 (10.9%)	30 (18–39)		23.93–36.07	0.098
	Patch	82 (89.1%)	21 (14–28)		18.58–23.42	
Blood cell count						
WBC	Decreased	4 (4.3%)	13(NE)		NE	0.493
	Normal	88 (95.7%)	22 (16–30)		19.71–24.29	
LY	Decreased	7 (7.6%)	24 (18–30)		18.87–29.13	0.818
	Normal	85 (92.4%)	22 (15–30)		19.00–25.00	
C-reactive protein						
Increased		11 (12.0%)	19 (13–28)		8.21–29.79	0.205
Normal		81 (88.0%)	22 (16–30)		19.65–24.35	

IQR = interquartile range; NE = not estimable.

**Table 3:** Cox proportional hazards regression for patient characteristics.

				Univariate			Multivariate		
				HR	95% CI	*P* value	HR	95% CI	*P* value
Age, yr									
≤20				1.305	0.48–3.57	0.604
21–40				1.316	0.69–2.50	0.403
41–60				1.235	0.70–2.17	0.462
>60				Ref
Coexisting conditions									
Cardiovascular cerebrovascular diseases			No	0.985	0.59–1.64	0.952
			Yes	Ref
Metabolic disease			No	1.17	0.51–2.68	0.712
			Yes	Ref
Duration time from symptom onset to start of TCM treatment, days									
*n* ≤ 7				7.674	4.49–13.11	<0.001	18.093	8.48–38.59	<0.001
7 < *n* ≤ 14				3.609	2.02–6.45	<0.001	5.804	3.00–11.23	<0.001
*n* > 14				Ref			Ref
Chief complaint at enrollment									
Fever			No	1.202	0.79–1.83	0.389
			Yes	Ref
Cough			No	1.423	0.93–2.19	0.108	4.101	1.36–12.39	0.012
			Yes	Ref	Ref
Dyspnea			No	1.600	0.86–2.97	0.137	4.271	1.80–10.12	0.001
			Yes	Ref			Ref
Respiratory symptoms			No	1.501	0.97–2.33	0.071
			Yes	Ref
Fatigue			No	1.618	0.70–3.74	0.261
			Yes	Ref
Chest CT									
Distribute		Multilobar		Ref
		Single		1.240	0.80–1.93	0.342
Density		GGO		Ref
				Consolidation		1.011		0.47–2.20	0.978
				Form		Diffuse		Ref
Patch		1.704	0.86–3.32	0.117
Blood cell count									
WBC	Decreased			Ref
	Normal			1.405	0.51–1.41	0.510
LY	Decreased			Ref
	Normal			1.092	0.50–2.38	0.824
C-reactive protein									
Increased				Ref
Normal				1.489	0.79–2.83	0.223

## Data Availability

The original data used to support the findings of this study are available from https://doi.org/10.6084/m9.figshare.14915121.v1.
